# Distributional Fate of Elements during the Synthesis of Zeolites from South African Coal Fly Ash

**DOI:** 10.3390/ma7043305

**Published:** 2014-04-23

**Authors:** Pieter W. Du Plessis, Tunde V. Ojumu, Ojo O. Fatoba, Richard O. Akinyeye, Leslie F. Petrik

**Affiliations:** 1 Department of Chemical Engineering, Cape Peninsula University of Technology, Keizersgracht and Tennant street, Cape Town 8000, South Africa; E-Mail: wynanddp@gmail.com; 2 Department of Environmental Nano Science, University of the Western Cape, Bellville 7535, South Africa; E-Mails: ofatoba@uwc.ac.za (O.O.F); richardakinyeye@gmail.com (R.O.A); lpetrik@uwc.ac.za (L.F.P)

**Keywords:** zeolite Na-P1, zeolite A, fly ash, material balance, X-ray diffraction, atomic emission spectrometry, X-ray fluorescence spectrometry

## Abstract

The synthesis of zeolites from South African coal fly ash has been deemed a viable solution to the growing economical strain caused by the disposal of ash in the country. Two synthesis routes have been studied thus far namely the 2-step method and the fusion assisted process. Fly ash contains several elements originating from coal which is incorporated in the ash during combustion. It is vital to determine the final destination of these elements in order to unveil optimization opportunities for scale-up purposes. The aim of this study was to perform a material balance study on both synthesis routes to determine the distributional fate of these elements during the synthesis of zeolites. Zeolites were first synthesized by means of the two synthesis routes. The composition of all raw materials and products were determined after which an overall and elemental balance were performed. Results indicated that in the 2-step method almost all elements were concentrated in the solid zeolite product while during the fusion assisted route the elements mostly report to the solid waste. Toxic elements such as Pb, Hg, Al, As and Nb were found in both the supernatant waste and washing water resulting from each synthesis route. It has also been seen that large quantities of Si and Al are wasted in the supernatant waste. It is highly recommended that the opportunity to recycle this liquid waste be investigated for scale-up purposes. Results also indicate that efficiency whereby Si and Al are extracted from fused ash is exceptionally poor and should be optimized.

## 1. Introduction

The use of coal fired power stations dates back to the 1880s over 100 years ago [1]. During the generation of electricity, the combustion of coal leads to the formation of incombustible solid residues. Of these residues, fly ash is the finest of the group of particulates and is produced on the largest scale [2]. In a developing country such as South Africa, where coal supplies are abundant, the use of coal as source of energy forms a core part of economic growth. However, the cost of managing the effects caused on the environment has become a nationwide concern.

In South Africa, a total of 36 Mt coal fly ash is produced annually from electricity generation alone [3]. On the average, 95% of the fly ash generated is disposed in ash dams and dumps [3]. The construction and maintenance of these dams requires large vacant land and has become a severe economic concern for the South African national power supplier. Once the land has been utilized for the disposal of fly ash, it is close to impossible to rehabilitate the soil in order to make it suitable for crops or any form of organic life [4]. The reason for this irreversible damage is due to the slow release of toxic elements from coal ash, as well as the changes in soil pH from the release of CaO [5–8]. Thus far, the only major use of fly ash has been as an additive in Portland cement [9]. The production of fly ash greatly outweighs the volumes required by the building industry thus limiting its use. With South Africa’s growing economy, it has become clear that a more sustainable approach must be investigated to relieve the environmental and economical strain caused by this waste product.

In numerous studies, it has been determined that the main constituents in fly ash are Al_2_O_3_, Fe_2_O_3_, SiO_2_ and CaO [2,10–12]. Due to the high concentrations of SiO_2_ and Al_2_O_3_ it is a suitable feedstock in the synthesis of zeolites as was first discovered by Höller and Wirsching in 1985 [13]. Since the first work done on the subject matter, various authors investigated the synthesis of a range of different zeolites from coal fly ash [14–18]. However, very little work has been done in an effort to use South African coal fly ash.

In recent endeavors, studies have been carried out with a view of synthesizing high quality zeolites from South African coal fly ashes [19,20]. In the studies performed to date, it has been possible to synthesize a range of zeolites using different South African coal fly ashes and different synthesis techniques. These zeolites include zeolite Na-P1, X, A, sodalite, cancrinite and analcime [19,21,22]. The use of fly ash as a feedstock in the synthesis of zeolites is a promising alternative to the current environmental predicament caused by its disposal. However, South African coals have been shown to contain various toxic elements such as As, Pb, Sb, Ba, V *etc.*, [23]. These elements are concentrated mostly in the fly ash during the combustion process due to the physical characteristics of this finer ash [24,25]. This aspect greatly complicates its use as a feedstock in zeolite synthesis. Details regarding the fate of these toxins during the synthesis process are not known. Environmental conservation is governed by strict legislation in South Africa such as the National Environmental Management: Waste Act 59 of 2008 [26].

Two principle processes have been used to synthesize zeolites with South African coal fly ashes. The first process consists of two steps namely alkaline aging of the ash followed by hydrothermal treatment [20,27,28]. The second synthesis route makes use of a pre-fusion step [21]. The fly ash is fused at high temperatures in order to dissolve the various components in the ash and generate soluble sodium silicates and aluminosilicates [17]. Thereafter the Si and Al components are extracted from the fused ash and hydrothermal treatment applied whereby zeolites are formed. Both processes generate wastes that would require disposal. Before these processes can be scaled up, the fate of the elements from the fly ash needs to be known. It is vital to determine whether the toxic elements from coal fly ash report to the liquid/solid wastes or the zeolite itself. This will enable environmental management plans to be set out for each process.

The aim of this study was to perform material balances around these zeolite synthesis processes in order to determine the distributional fate of the elements originating from the coal fly ash.

## 2. Results and Discussion

### 2.1. Elemental Composition of Coal Fly Ash

Table 1 illustrates the major oxides and trace elements in Arnot fly ash as presented by X-Ray Fluorescence spectroscopy analysis (XRF). Fly ash is classified into two broad groups namely class F and C according to the ASTM standard C618-95 [29]. The fly ash used in this study was classified as class F ash, whereby the SiO_2_ + Al_2_O_3_ + Fe_2_O_3_ mass exceeds 70% of the total fly ash mass (Table 1). The major elements found in the ash were Si and Al, which are the two main elements of which zeolites are composed. The ratio of SiO_2_/Al_2_O_3_, a factor greatly influencing the mechanism of zeolite formation [30], was found to be 1.76 [30]. Amongst the trace elements, the most concentrated elements were found to be Sr, Ce and Ba. It is clear that the toxic elements found in South African coals [23] are concentrated in the fly ash during the combustion process.

By performing both overall and elemental material balances, the distribution of these elements throughout the zeolite synthesis process was tracked. The final destination of toxic elements will greatly affect the disposal costs and pre-treatment requirements as set forth in South African legislation [26].

### 2.2. Synthesis and Material Balance Using the 2-Step Synthesis Method

**Figure 1.**
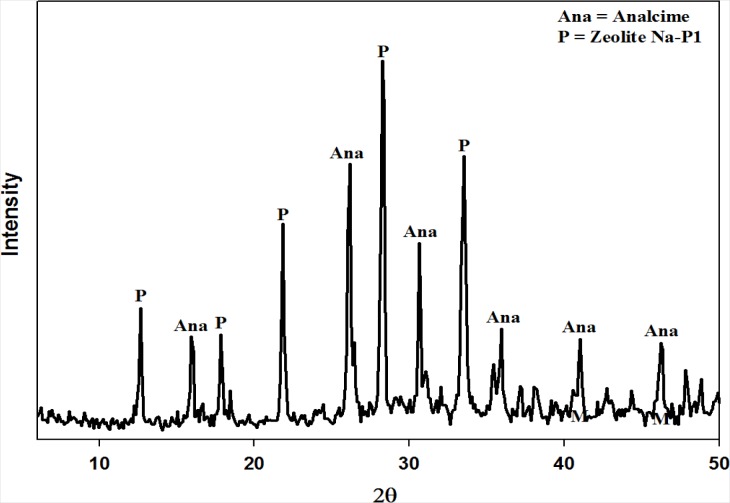
X-Ray powder diffraction (XRD) patterns illustrating the two zeolite crystal products produced by applying the 2-step method as synthesis approach.

Figure 1 below illustrates the X-Ray powder diffraction (XRD) results obtained after synthesizing zeolites from fly ash using the 2-step approach. Two main zeolite products were obtained namely analcime and Na-P1. Unreacted fly ash and other minor products, not visible from XRD, were also present in the solid product. These results are comparable to those obtained by Mainganye [31] when applying the same operating conditions. With the authors’ results successfully reproduced the next steps of the material balance approach were instigated.

**Figure 2.**
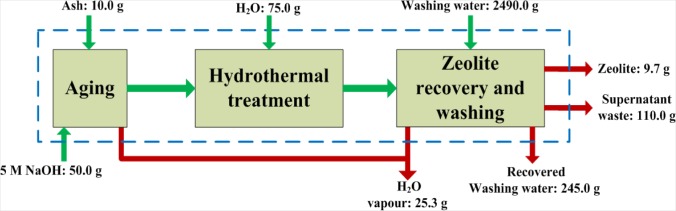
Block flow diagram illustrating the overall mass balance of the 2-step method.

The basis of the material balances performed over this synthesis process was taken as 10 g of fly ash feed. Figure 2 illustrates a simple block flow diagram of the synthesis process with the respective weights of material crossing the system boundary. From this overall material balance it was seen that from 10 g of fly ash, on average, 9.7 g of dry zeolite product was obtained. The water used to wash the zeolite products could be recovered effectively through filtration. From the 2500 mL ultrapure water used to wash the zeolite product, 2490 mL liquid could be recovered. On average 110 g of liquid supernatant waste resulted from the synthesis process. The overall balance also revealed that 25.3 g of water losses resulted from the process. These losses were due to evaporation at two main stages in the process. The aging step was performed in an open reactor which allowed vapor to escape. It is recommended that an improved (sealed) reactor design should be considered for this stage. The second step where water loss occurs is during drying of the zeolite product in a hot air oven.

Table 2 illustrates the weight percentage (wt%) distribution of elements from fly ash amongst the various products and wastes generated during the synthesis process. As can be seen from Table 2, most of the Si and Al from fly ash, 72.2% and 81.5% respectively, reports to the zeolite product. Nearly 50% of the K from the fly ash reported to the zeolite product which could possibly have as a competing charge stabilizing ion [32]. At this point in the investigation, it was not clear whether the K was incorporated in the zeolite pores or merely in the solid product as a whole. The values for Na in Table 2 takes into account the total Na input into the system, *i.e.*, from fly ash and the 5 M NaOH solution. A mere 12.4% of Na incorporated into the zeolite product points to a great wastage of NaOH in the system. The possibility of recycling waste streams containing this product needs to be investigated. With 23.7% of the Si and 15.8% Al still left in the supernatant waste, there is room for improvement in conversion efficiency. It was found that most elements in the fly ash remain in the solid zeolite product. From the list of elements, 100% of Mn, Mg, Ca, Ti, S, Ba, Ce, Co, Cu, Sr, Y and Zn were found in the synthesized product. Although it is not known what effect this has on the application of the zeolite products, it does make disposal of the spent material less complicated. It has been shown that the elements originating from fly ash show relatively low mobility when included in the solid products [33]. The only elements found in significant quantities in the supernatant waste were Si, Al, Fe, Na, K, P, Ni, Pb, Rb and V. The presence of these elements complicates the disposal of the liquid waste. Elements such as Pb, Nb and Al are of great concern due to their toxic nature. Treatment and disposal of this liquid waste would be an expensive process operation, which greatly questions the feasibility of the initiative to synthesize zeolites from coal fly ash. A recent study has shown that protocols can be developed whereby liquid waste can be recycled in the 2-step process [34]. However, recycling the liquid waste yields even less quantities of zeolite Na-P1 due to the accumulation of Si in the waste [34]. Traces of two other highly toxic elements, namely As and Hg were found in the supernatant waste. These two elements were not included in the material balance since they could not be detected through XRF analysis. The average concentration of As and Hg were found to be 0.5 ppm and 0.2 ppm respectively. The water recovered from washing the zeolite products has also revealed some levels of contamination. The main elements of concern are the toxic elements such as Pb, Nb and Al. Although a large weight fraction of these three elements reports to the liquid waste, their concentrations are very low due to the volumes of washing water produced. The concentrations of Pb, Nb and Al were found to be 0.056, 0.075 and 0.020 ppm respectively. However, greater levels of Hg were also found in the washing wastewater. The average concentration of Hg was 0.4 ppm. Although all these exist in low concentrations, it will still complicate its disposal. Alternative zeolite washing methods need to be investigated to avoid generating waste containing these three highly toxic elements.

### 2.3. Synthesis and Material Balance Using the Fusion Assisted Synthesis Method

**Figure 3.**
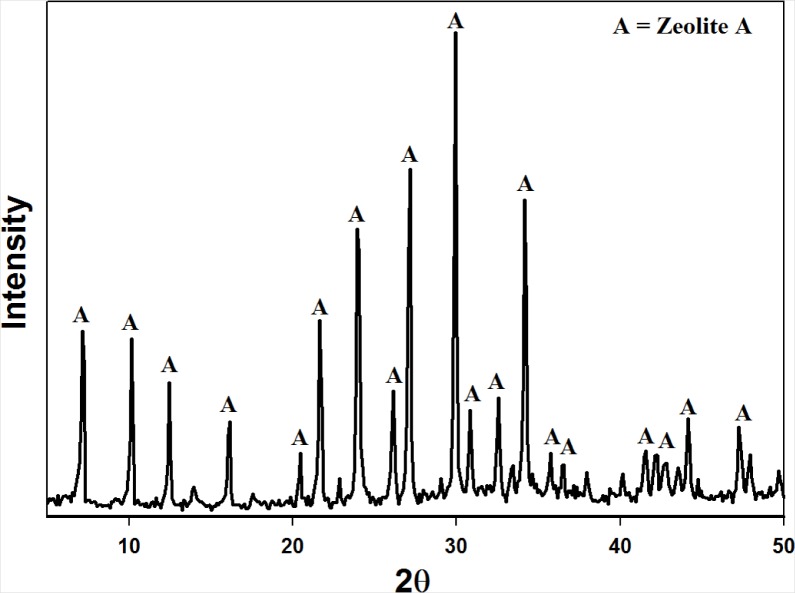
XRD powder diffraction pattern illustrating zeolite A product obtained by means of the fusion assisted process.

**Figure 4.**
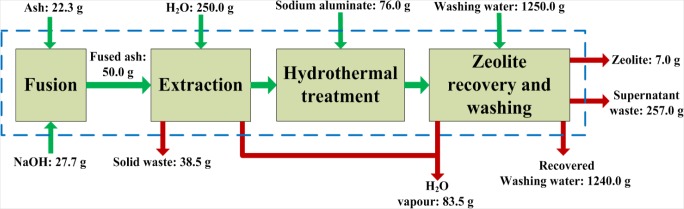
Block flow diagram illustrating the overall mass balance of the fusion assisted method.

Figure 3 illustrates the XRD pattern obtained for the zeolite product synthesized by applying the fusion assisted method. The results indicate that a highly crystalline pure phase zeolite A was produced. These results are similar to those obtained by Musyoka [21] proving the reproducibility of the authors’ results. Figure 4 illustrates the overall material balance performed over this synthesis approach. The basis of the material balance was taken as 50 g of fused fly ash. The fused ash consequently consisted of 22.3 g raw fly ash and 27.7 g analytical grade NaOH powder. After the extraction step the solid waste (sludge) resulting from the process was dried and on average weighed 38.5 g. The final zeolite product was washed with 1250 g ultrapure water out of which an average of 1240 g could be recovered. The supernatant waste separated from the zeolite product totaled 257 g. However, an average of 7.0 g of zeolite A was synthesized per run. This overall yield is markedly poor relative to the 2-step process. On the other hand the 2-step process is time consuming and less robust. It is clear that both synthesis approaches have certain drawbacks and advantages over each other.

The wt% distribution of elements amongst the different liquid and solid products is tabulated in Table 3. A mere 19.1% of the Si originating from the fly ash reported to the zeolite product as opposed to the 72.2% of the 2-step process. This was due to the fact that most Si was lost in the solid waste after extraction of clear solution from the fused ash. This is the main reason why a mere 7.0 g zeolite yield was obtained. To improve the yield a new approach needs to be investigated towards extracting optimum amounts of Si and Al from the fused ash to reduce wastage. The wt% of Al takes into account the Al input from both fly ash and the sodium aluminate solution. In this case, most Al reported to the solid waste instead of the zeolite crystal. Other than these two critical elements, it was found that almost all elements originating from the fly ash reported to the solid waste. This was also seen in the 2-step synthesis approach where the solids were incorporated in the overall solid/zeolite product.

Out of the list of elements, 100% of Ce, Co, Cu and Y were found in the solid waste. Also nearing 100% concentration in the solids were Fe, Mn, Mg, Ca, Ti, Ba, Rb and Sr. The zeolite product, similar to the 2-step process, contained a significant amount of the K originating from fly ash. The fusion assisted process makes use of only the clear solution extracted from fused ash and not the solid waste. Therefore it can be assumed that the K^+^ ions are incorporated in the zeolite pores as charge stabilizing ions in both synthesis approaches. It was also seen that in the fusion assisted process, 74.0% of the sulfur reports to the zeolite product. This was believed to occur due to adsorption of sulfur onto zeolite A. The capability of zeolite A to adsorb sulfur in various forms was illustrated by Steijns and Mars [35]. In the supernatant waste, large fractions of the Si, Al and Na were found resulting in a great loss of these elements. Phosphorous and Vanadium were concentrated in the supernatant waste as also seen in the 2-step process. The toxic element Ba was also included in the supernatant waste contrary to the 2-step process. Other toxic elements such as As, Hg, Pb, Rb and Al were also found in the supernatant and washing water waste. Of particular concern is the large fraction of lead (42.1%) reporting to the washing water waste. Niobium (41.8%) was found in the washing water waste, as opposed to the 16.2% of the 2-step process. This element forms part of the list of rare earth elements (REE) and its extraction from the liquid could yield promising benefits.

## 3. Experimental Section

### 3.1. Research Approach

In order to determine the fate of elements originating from coal fly ash during the synthesis of zeolites, a generalized approach was formulated as illustrated below:

(1) Identification of the compositional characteristics of raw fly ash feed.
(2) Determination of the basis for the material balance study.
(3) Synthesis of zeolites and investigation of the reproducibility of results obtained by Musyoka [21] and Mainganye [19].
(4) Determination of the overall material balance by measuring weights of all feeds, products and wastes.
(5) Determination of the elemental composition of all feeds, products and wastes.
(6) Performing the elemental balance to determine the distributional fate of elements in the system.

### 3.2. Zeolite Synthesis

#### 3.2.1. Two-Step Alkaline Activation Method

**Figure 5.**
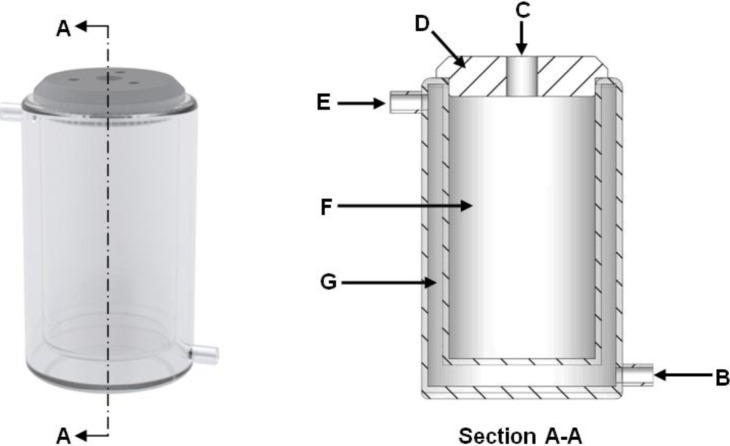
Double walled glass reactor used during the aging step. (A-A) Section cutout view; (B) Hot water inlet; (C) Cutout in reactor lid allowing the impeller shaft to pass through; (D) Reactor lid; (E) Hot water outlet; (F) Reaction volume; (G) Heating/cooling water space surrounding the inner reactor wall.

The zeolite synthesis procedure was adopted from Musyoka [28] which consists of two steps, *i.e.*, aging followed by hydrothermal treatment. The aging step was performed in a 100 mL double walled glass reactor (Figure 5). The reactor was connected to a variable temperature water bath which maintained the aging medium at 47 °C. First 50 mL 5 M NaOH solution was prepared and preheated inside the 100 mL glass reactor. Once the NaOH solution reached the required temperature, the aging step was initiated with the addition of 10 g of coal fly ash to the heated solution. The aging medium was mixed utilizing a 4-blade paddle impeller at 200 rpm as recommended by Mainganye [19]. The aging step then proceeded for 48 h. After the aging step, 75 mL of ultrapure water was added to the aged medium under agitation, after which the mixture was transferred into Teflon lined autoclave reactors. The autoclave reactor used was the 23 mL general purpose digestion vessel from the Parr Instrument Company (Model number: 4745). The autoclave reactors were placed in a hot air oven at 140 °C in order for the hydrothermal treatment stage to commence. The hydrothermal stage proceeded for 48 h after which the solid zeolite product was separated from the liquid waste supernatant through filtration. The zeolite product was then washed with 2500 mL ultrapure water and dried in a hot air oven at 80 °C.

#### 3.2.2. Fusion Assisted Synthesis

**Figure 6.**
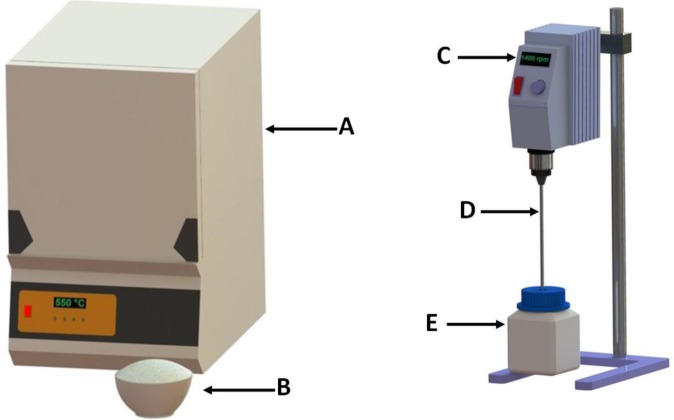
Experimental setup illustrating the fusion of ash and extraction of Si and Al from fused ash. (A) Electrical furnace; (B) Mixture of fly ash and NaOH powder in a crucible; (C) Overhead stirrer set at 1400 rpm; (D) 4-blade paddle impeller; (E) Rectangular mixing vessel.

**Figure 7.**
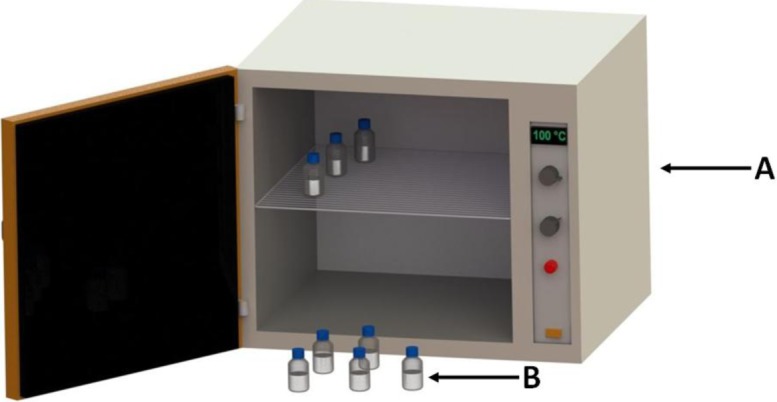
Experimental setup illustrating the hydrothermal treatment step whereby zeolite A crystals are formed. (A) Hot air oven; (B) 250 mL glass bottles containing adjusted clear solution ready for hydrothermal treatment.

The method used to produce zeolite A from South African ash was adopted from work done by Musyoka in 2012 [21]. The process starts off by mixing coal fly ash with crushed analytical grade sodium hydroxide in a mass ratio of 1:1.2 (ash: NaOH). The mixture of NaOH and fly ash was then fused at 550 °C for 90 min in an electrical furnace (Figure 6). The fused ash was allowed to cool to ambient temperature and then ground into a fine powder using a mortar and pestle. Then 50 g of the ground ash and 250 mL ultrapure water were added to a rectangular plastic mixing vessel (Figure 6). The mixture was closed and agitated with an overhead stirrer equipped with a 4-blade paddle impeller mixing at a speed of 1400 rpm for 120 min to allow extraction of Si and Al into solution. The mixing vessel was not equipped with baffles as this was not necessary due to the fact that the corners of the vessel broke the tangential flow [36] inducing a baffling effect which improved mixing. The mixed slurry was then filtered and the clear solution’s Si/Al adjusted by the addition of 0.59 M sodium aluminate solution in a volume ratio of 5:2 (clear solution: sodium aluminate solution). The mixture was agitated using a magnetic stirrer until a milky suspension started to form. The aluminate solution was made up by dissolving analytical grades sodium aluminate and sodium hydroxide in separate batches of ultrapure water. The NaOH and NaAlO_2_ were dissolved in ultrapure water in mass ratios of 50:4.8 (ultrapure water: NaOH) and 50:2.4 (ultrapure water: NaAlO_2_) respectively. The two solutions were then added together and mixed using a magnetic stirrer for 30 min. The milky solution was then transferred into 250 mL glass bottles in allocates of 100 mL per bottle. The solutions were then subjected to hydrothermal treatment by placing the glass bottles in a hot air oven for 120 min at 100 °C (Figure 7). The products were then separated by filtration and washed with ultrapure water. The washed zeolite product was dried at 80 °C in a hot air oven after which it was crushed and stored in airtight containers.

### 3.3. Materials and Characterization Techniques

The coal fly ash used in this study was collected from a power station situated in Mpumalanga, South Africa. Analytical grade sodium hydroxide pellets were used for the preparation of 5 M NaOH solutions and sodium aluminate solutions. Ultrapure water was use in all water applications during the process which includes the preparation of all solutions, water addition step during the 2-step method and washing of zeolite products. The elemental composition of all solid material; including raw fly ash, zeolite products and solid waste; was determined by performing XRF spectroscopy. On the other hand, elemental concentrations in liquid products and wastes were determined with inductively coupled plasma atomic emission spectrometry (ICP-AES). The mineralogy of zeolite products was determined by means of XRD using Cu-Kα radiation in a range of 4 < 2θ < 60. Zeolite structural information was determined by means of attenuated total reflectance Fourier transform infrared spectroscopy (ATR-FTIR).

## 4. Conclusions

Material balances were performed on two process routes whereby zeolites are synthesized from South African coal fly ash. The various elements originating from coal fly ash were tracked throughout the processes to determine their distributional fate. In the 2-step process, the majority of the elements report to the solid zeolite product, while in the fusion assisted process nearly all elements concentrated in the solid waste. Both processes generated liquid wastes contaminated with highly toxic elements. Amongst these elements were found As, Pb, Hg, Al and Nb. Disposal of these toxic wastes will create severe environmental and economical problems. It is recommended that the possibility to recycle the liquid supernatant waste be investigated. Also, alternative zeolite washing approaches needs to be considered to avoid the production of large volumes of washing water waste. It was found in both synthesis approaches that vanadium (±50%) and phosphorous (75.5%–97%) mostly reports to the supernatant and washing water waste. The yield efficiency of the fusion assisted synthesis approach was found to be very poor. A mere 19.6% of Si and 21.6% Al from the fly ash were incorporated into the zeolite product. It is recommended that the extraction of Si and Al from fused ash be optimized in order to minimize wastage of these two elements in the solid waste. Also, in this synthesis route, it was discovered that niobium was concentrated in the liquid waste. Being a rare earth element, recovery of this element might be an advantageous task to pursue.

## Figures and Tables

**Table 1.** X-ray fluorescence results of Arnot coal fly ash illustrating the quantities (wt%) of the major oxides and trace elements (ppm) of which it is composed.

Major oxides	Mean wt%	Trace elemental	Concentrations (ppm)
SiO_2_	55.44	Ba	486
Al_2_O_3_	31.51	Ce	254
Fe_2_O_3_	4.94	Co	30
MnO	0.03	Cu	110
MgO	1.18	Nb	37
CaO	3.76	Ni	125
Na_2_O	0.04	Pb	90
K_2_O	0.47	Rb	56
TiO_2_	1.11	Sr	989
P_2_O_5_	0.30	V	79
SO_3_	0.06	Y	94
Loss On Ignition	1.22	Zn	135
SiO_2_/Al_2_O_3_	1.76	–	–

**Table 2.** Elemental balance illustrating the distribution of the elements (wt%) originating from fly ash amongst the different products resulting from the 2-step synthesis method.

Element	Zeolite product	Supernatant waste	Washing water
Si	72.2%	23.7%	4.1%
Al	81.5%	15.8%	2.7%
Fe	86.5%	11.4%	2.1%
Mn	100.0%	0.0%	0.0%
Mg	100.0%	0.0%	0.0%
Ca	100.0%	0.0%	0.0%
Na	12.4%	45.2%	42.3%
K	49.4%	50.6%	0.0%
Ti	100.0%	0.0%	0.0%
P	3.2%	78.9%	17.9%
S	100.0%	0.0%	0.0%
Ba	100.0%	0.0%	0.0%
Ce	100.0%	0.0%	0.0%
Co	100.0%	0.0%	0.0%
Cu	100.0%	0.0%	0.0%
Nb	80.7%	3.2%	16.2%
Ni	64.5%	35.5%	0.0%
Pb	63.2%	21.4%	15.5%
Rb	78.2%	17.9%	3.9%
Sr	100.0%	0.0%	0.0%
V	50.8%	49.2%	0.0%
Y	100.0%	0.0%	0.0%
Zn	100.0%	0.0%	0.0%

**Table 3.** Elemental balance illustrating the distribution of the elements (wt%) originating from fly ash amongst the different products resulting from the fusion assisted synthesis method.

Element	Solid waste	Zeolite product	Supernatant waste	Washing water
Si	66.2%	19.6%	9.5%	4.7%
Al	68.7%	21.6%	7.9%	1.8%
Fe	98.8%	0.3%	0.2%	0.7%
Mn	87.9%	0.0%	9.3%	2.7%
Mg	97.2%	0.0%	0.6%	2.2%
Ca	97.2%	0.0%	0.7%	2.2%
Na	26.3%	4.8%	33.9%	35.0%
K	23.5%	21.2%	40.5%	14.7%
Ti	99.6%	0.2%	0.1%	0.2%
P	24.5%	0.0%	63.8%	11.6%
S	26.0%	74.0%	0.0%	0.0%
Ba	89.4%	2.4%	3.2%	5.0%
Ce	100.0%	0.0%	0.0%	0.0%
Co	100.0%	0.0%	0.0%	0.0%
Cu	100.0%	0.0%	0.0%	0.0%
Nb	54.3%	0.0%	3.9%	41.8%
Ni	78.5%	0.0%	21.5%	0.0%
Pb	51.1%	0.0%	6.8%	42.1%
Rb	89.3%	0.0%	10.7%	0.0%
Sr	99.6%	0.0%	0.0%	0.4%
V	45.2%	0.0%	39.6%	15.3%
Y	100.0%	0.0%	0.0%	0.0%
Zn	53.8%	3.6%	11.9%	30.8%
